# Phylogenetic relatedness and host plant growth form influence gene expression of the polyphagous comma butterfly (*Polygonia c-album*)

**DOI:** 10.1186/1471-2164-10-506

**Published:** 2009-10-31

**Authors:** Hanna M Heidel-Fischer, Dalial Freitak, Niklas Janz, Lina Söderlind, Heiko Vogel, Sören Nylin

**Affiliations:** 1 Department of Entomology, Max Planck Institute for Chemical Ecology, Hans-Knöll-Straße 8, D-07745 Jena, Germany; 2 Department of Zoology, Stockholm University, 106 91 Stockholm, Sweden

## Abstract

**Background:**

The mechanisms that shape the host plant range of herbivorous insect are to date not well understood but knowledge of these mechanisms and the selective forces that influence them can expand our understanding of the larger ecological interaction. Nevertheless, it is well established that chemical defenses of plants influence the host range of herbivorous insects. While host plant chemistry is influenced by phylogeny, also the growth forms of plants appear to influence the plant defense strategies as first postulated by Feeny (the "plant apparency" hypothesis). In the present study we aim to investigate the molecular basis of the diverse host plant range of the comma butterfly (*Polygonia c-album*) by testing differential gene expression in the caterpillars on three host plants that are either closely related or share the same growth form.

**Results:**

In total 120 genes were identified to be differentially expressed in *P. c-album *after feeding on different host plants, 55 of them in the midgut and 65 in the restbody of the caterpillars. Expression patterns could be confirmed with an independent method for 14 of 27 tested genes. Pairwise similarities in upregulation in the midgut of the caterpillars were higher between plants that shared either growth form or were phylogenetically related. No known detoxifying enzymes were found to be differently regulated in the midgut after feeding on different host plants.

**Conclusion:**

Our data suggest a complex picture of gene expression in response to host plant feeding. While each plant requires a unique gene regulation in the caterpillar, both phylogenetic relatedness and host plant growth form appear to influence the expression profile of the polyphagous comma butterfly, in agreement with phylogenetic studies of host plant utilization in butterflies.

## Background

Chemical defenses of plants influence the host range of herbivorous insects [1-4]. Although by no means the only factor involved in shaping insect-host associations [5], few researchers would argue against its general importance. However, there is an ongoing argument over the reasons for similarities among plant defense chemicals. There are two ways for evolution to achieve similarity: either through shared ancestry or through evolutionary convergence (or parallelism). Ehrlich and Raven [2] suggested that related insects tend to feed on related plants, and several other studies have continued to demonstrate a role of host plant phylogeny (shared ancestry) on patterns of host plant use [6-14]. Hence, there is strong support for a historical component in patterns of host plant use.

On the other hand, several authors have also pointed out that plant chemistry does not always follow phylogeny [10,15,16]. Feeny [17] suggested that plant defense strategies should differ depending on their "apparency"; plants that are long-lived and/or physically large will always be found by attacking insects and should possess constitutive chemical defenses such as tannins, terpenes, and flavanoids (see also [18-21]) that have a quantitative, dosage-dependent effect. Unapparent plants, with lower risk of detection by herbivores, should instead tend to utilize induced chemical defenses. Trees should be the most apparent of plants as they are both physically large and long-lived. According to the apparency hypothesis, trees should then tend to have more convergent constitutive defenses than herbs, and as a consequence we should see more host shifts involving trees than herbs. Despite some criticism and uncertainty regarding the mechanisms involved, the apparency theory has remained a useful framework for thinking about the evolution of plant chemical defense in the context of plant community heterogeneity [22].

In a phylogenetic reanalysis of Ehrlich and Raven's [2] study on butterfly and plant coevolution, Janz & Nylin [9] found strong effects of both plant phylogeny and growth form on patterns of host use among butterflies. An overwhelming majority of host shifts occurred while feeding on trees, giving support for Feeny's "plant apparency" hypothesis. Trees appeared to serve as a "bridge" that could facilitate host shifts between distantly related plants.

Hence, there is support for both shared ancestry and convergent evolution in the large-scale chemical structuring of insect-host associations, but the mechanistic basis remains largely unknown. However, recent years have seen great progress in understanding of the molecular mechanisms that enable insect to feed on certain host plants. In general it is assumed that insects apply phase I and phase II detoxifying enzymes to metabolize secondary plant compounds. Several studies have for example revealed the important role of the cytochrome P450 enzyme family for detoxification of plant secondary compounds as well as insecticides. [23-27]. Glutathione-S-transferases (GSTs) have also been shown to be induced in generalist and specialist lepidopteran larvae upon feeding on their host plants [28,29]. Wittstock et al. [30] identified the Nitrile-specifier Protein (NSP) in *Pieris rapae*. NSP redirects the glucosinolate hydrolysis and by that enables the Pierinae butterflies to feed on the plant family Brassicaceae. Further research has been done on the evolution of NSP showing its evolution by domain and gene duplication from a gene of unknown function that is widespread in insect species [31,32]. *Plutella xylostella *is also feeding on glucosinolate containing plants. Here the Glucosinolate sulfatase (GSS) inhibits the hydrolysis of glucosinolates completely by forming a desulfo-glucosinolate. In spite of progress in recent years, much is still to be discovered in the detoxification mechanisms of insects.

In the present study we aim to investigate the molecular basis of the diverse host plant range of the comma butterfly (*Polygonia c-album*, Lepidoptera: Nymphalidae). *P. c-album *is a widespread polyphagous butterfly species of the family Nymphalidae. It is found all over Eurasia, from England to Japan and from the central of Sweden to the northern tip of Africa. The larvae can be found on host plants from several taxa: the "urticalean rosids" *Urtica, Humulus *and *Ulmus *and the distantly related *Salix *(Salicaceae), *Ribes *(Grossulariaceae), *Betula *and *Corylus *(Betulaceae) [33]; hence the species is at the extreme end of polyphagy among butterflies, although by no means an indiscriminate generalist [9].

For this study, we used a test array with three host plants of *P. c-album *that are either closely related (Stinging nettle *Urtica dioica *and Wych Elm *Ulmus glabra *- both in Urticales) or share the same growth form (Great Sallow *Salix caprea *and *Ulmus glabra *- both trees). Following Feeny's "plant apparency" hypothesis and the phylogenetic patterns of host shifts found by Janz & Nylin [9] and Janz et al. [8], we expected to find more similarities in the gene expression profiles of caterpillars that have been feeding on plants that either have a shared ancestry (*U. dioica *and *U. glabra*) or belong to the same growth form (*S. caprea *and *U. glabra*).

## Results

### General patterns of gene expression

**Figure 1:**
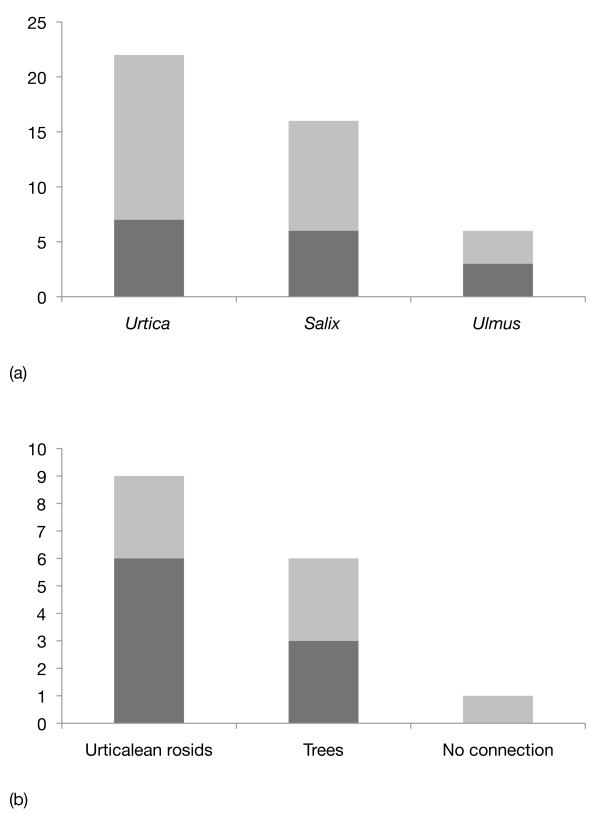
**General patterns of gene expression in the larval midgut of *Polygonia c-album***. Dark grey represent qualitative differences or similarities (present/absent), light grey represent quantitative differences or similarities (more/less). **(a) **The number of uniquely upregulated genes on the three host plants used in the study, showing that significantly fewer genes were uniquely upregulated on *Ulmus*. **(b) **Pairwise similarities in upregulation between two of the three host plants. "Urticalean rosids" = *Urtica dioica *+ *Ulmus glabra*; "Trees" = *Ulmus glabra *+ *Salix caprea*; "No connection" = *Urtica dioica *+ *Salix caprea*. Following predictions, more genes were concurrently upregulated on plants that shared either ancestry or growth form.

The pattern of expression in the midgut corresponded to the formed predictions that more similarities will be found in the gene expression profiles of caterpillars that have been feeding on plants that either have a shared ancestry (*U. dioica *and *U. glabra*) or belong to the same growth form (*S. caprea *and *U. glabra*) (Additional File 1; Table S1, Figure 1). There were significantly more uniquely upregulated genes in the larval midgut on the *Urtica *and *Salix *diets than on the *Ulmus *diet (likelihood-ratio χ^2 ^test, observed frequencies (*Urtica, Salix, Ulmus*) = 22, 16, 6 (expected frequencies equal), χ^2 ^= 9.8990, p = 0.007). Pairwise similarities in upregulation were higher between plants that shared either growth form or were phylogenetically related (likelihood-ratio χ^2 ^test, observed frequencies (Urticalean rosids, trees, no connection) = 9, 6, 1 (expected frequencies equal), χ^2 ^= 7.4839, p = 0.024).

**Figure 2:**
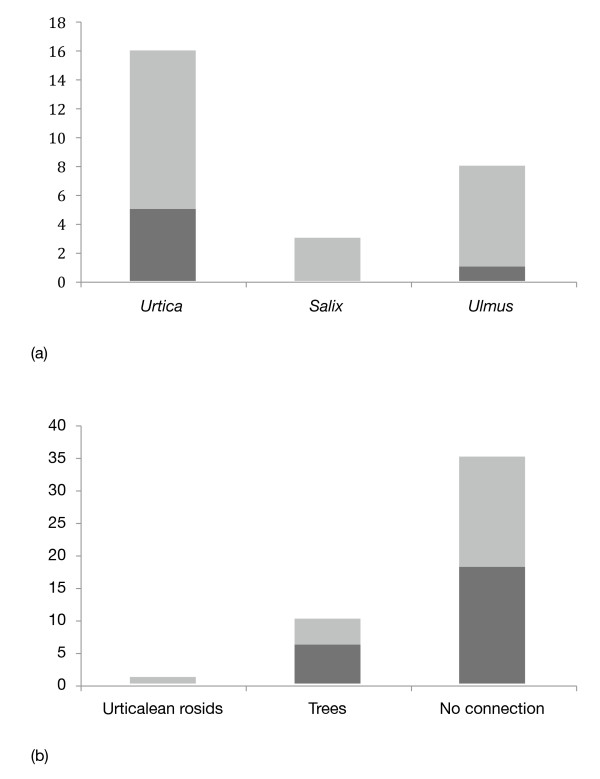
**General patterns of gene expression in the larval restbody of *Polygonia c-album***. Dark grey represent qualitative differences or similarities (present/absent), light grey represent quantitative differences or similarities (more/less). The pattern seen in the restbody is distinctly different from the midgut. **(a) **The number of uniquely upregulated genes on the three host plants used in the study. **(b) **Pairwise similarities in upregulation between two of the three host plants. "Urticalean rosids" = *Urtica dioica *+ *Ulmus glabra*; "Trees" = *Ulmus glabra *+ *Salix caprea*; "No connection" = *Urtica dioica *+ *Salix caprea*. Again, the pattern is different from the midgut; there are no effects of shared ancestry or growth form.

There were no corresponding patterns in the restbodies (Additional File 1; Table S2, Figure 2). Here, more genes were uniquely upregulated on *Urtica *and *Ulmus *than on *Salix *(likelihood-ratio χ^2 ^test, observed frequencies (*Urtica, Salix, Ulmus*) = 16, 3, 8 (expected frequencies equal), χ^2 ^= 9.9355, p = 0.007) and there were more similarities in upregulated genes between the "unrelated" plants than between the trees or urticalean rosid groups (likelihood-ratio χ^2 ^test, observed frequencies (Urticalean rosids, trees, no connection) = 1, 10, 35 (expected frequencies equal), χ^2 ^= 43.7634, p < 0.001).

### Function and patterns of differentially expressed genes

In total we identified 120 differentially expressed genes, 55 in the midgut of *P. c-album *and 65 in the restbody (Additional File 1; Table S1 and Table S2). In the midgut six sequences gave no hit and in the restbody 2 sequences gave no hit in BLAST searches. Many of the differentially expressed genes in the midgut are likely to be involved in metabolism and digestion, ranging from protein degradation to starch and lipid breakdown for nutrient acquisition. We could also identify eight ribosomal genes and seven genes that are involved in translation regulation and the DNA structure in the cell nucleus. Furthermore, we found three genes involved in immunity and one transmembrane transport domain. There were also differentially expressed proteases, but we did not identify any differentially expressed known detoxifying enzymes in the midgut. Four of the differentially expressed genes were unknown (Additional File 1; Table S1).

In the restbody of the comma butterfly larvae again, the majority of the gene products are most likely involved in the metabolism of the caterpillar or translate into ribosomal proteins. Seven of the sequenced gene fragments appear to have a structural role, and are mostly involved in chitin binding. We could also identify translation regulation genes and some involved in cellular architecture (Additional File 1; Table S2). One sequence showed moderate similarities (e-value of 0,00000008) to potential detoxification genes, namely a cytochrome P450 of *Plutella xylostella *(Additional File 1; Table S2).

### Confirmation of differentially expressed genes

Of the total 122 genes originating from GeneFishing, we picked 27 genes (18 from midguts and 9 from restbodies) to confirm differential gene expression pattern with qRT-PCR. We were able to see identical expression patterns in the GeneFishing and in the qRT-PCR results for 14 of the 27 genes (10 in midguts and 4 in restbodies). Partial similarity (same in relation to one diet) in expression patterns between two independent methods could be observed in 7 genes (4 in midguts and 3 in restbodies) and 5 genes (4 in midguts and 1 in restbodies) behaved differently (Additional File 1; Table S3).

In the midgut, genes that showed similar expression patterns as in the GeneFishing experiment included proteins involved in digestion, namely chymotrypsinogen-like protein 3, serine protease, chymotrypsin-like protease, alpha-amylase, trypsin-like protease, trypsin Ia precursor, long-chain acyl-CoA synthetase and short-chain dehydrogenease/reductase 2. In addition, also a ribosomal protein S16 and the immune response related protein cobatoxin showed same expression patterns in the qRT-PCR and in the differential gene expression study. In the restbodies alpha-amylase and the serine protease precursor were similarly expressed. The stress related heat shock cognate 70 protein and a potential cytochrome P450 also showed similar expression pattern in both methods.

## Discussion

Our expression data suggest a complex interaction between the comma butterfly and its host plants. On the one hand, each plant species appears to require a very specific subset of genes to be regulated in the midgut upon feeding. This involves digestion proteins, immunity related genes, general metabolism genes and ribosomal protein as well as translational and transport genes. In the restbody we also find cell signaling related domains, hormone synthesis genes and genes involved in silk production, suggesting a gene regulation, tuned specifically for each plant species. On the other hand, counting of upregulation of genes in the midgut shows a suggestive pattern (Figure 1). Both species of the Urticales family (*Ulmus *and *Urtica*), as well as both trees, have a higher agreement of gene regulation than do the stinging nettle and the great sallow. Especially the digestion and ribosomal genes show clear differences here (Additional File 1; Table S1). This suggests that phylogenetic and/or growth form relatedness demand more similar expression profiles in the midgut of the caterpillars.

However, this pattern only holds true for the midgut of the comma butterfly; the restbody of the caterpillars show a completely different picture. Here the stinging nettle and great sallow diet in contrast share the highest similarity in gene expression, while the wych elm appears to require less gene upregulation in general (Additional File 1; Table S2, Figure 2). The midgut is the place of contact with the food bolus, and the location where the first detoxifying and digestive actions will take place, whereas the restbody is only indirectly involved in this process by receiving the solubilized products and only sometimes toxic or toxin degradation products. Here the actual nutrient content is of more importance. This might explain the converse picture we observe in those two tissues. While the defense compounds of the phylogenetically or growth form-related plants might be more similar, our data suggests that the nutritional value does not follow this line.

**Figure 3:**
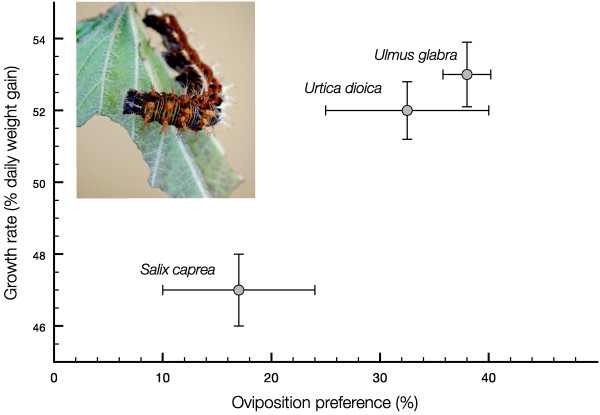
**Correlation between preference and performance in *Polygonia c-album***. Correlation between relative oviposition preference (degree of preference in simultaneous choice trials) and larval performance (mean growth rate) for the plants used in the present study (means ± SE). Data from [34].

Previous data on larval performance show that caterpillars of *P. c-album *perform differently on the plant species used in this study, suggesting different levels of adaptation and/or different nutritional values (e.g. [34]). The general pattern is that the plants in Urticales (*U. dioica *and *U. glabra*) support the highest growth rates, while larvae grow slower on *S. caprea *(but pupal weights are typically larger). These differences are also reflected in the preference hierarchy of the ovipositing females, which means that the preference-performance correlation is good on a population level [33,34] (Figure 3).

Oviposition preference and larval performance on *U. glabra *are more variable between years than the other plants, so that in some years it is considerably less preferred than *U. dioica*, with a correspondingly lower performance (Nylin, S. and Janz, N., unpublished data). This could be caused by higher between-year variability in *U. glabra *compared to the other two hosts. Such unpredictable variation would make it more difficult to adapt to the host. Indeed, the lower amount of uniquely upregulated genes on *Ulmus *found in this study would suggest such insufficient adaptation to this host.

In the midgut we could not detect any differentially expressed known detoxifying genes, such as cytochromes P450s or glutathione-S-transferases (GST). However, those enzymes are with high probability active in the gut of the comma butterfly larvae when encountering plant material. The GeneFishing method identifies solely differentially expressed genes. Hence, *P. c-album *might very well express such Phase I and Phase II detoxifying genes, but in a constant manner independent of the diet. Being a highly polyphagous insect species, the comma butterfly might be endued with very broad acting detoxifying enzymes that can cope with a wide variety of the compounds and hence are expressed continuously when feeding independent of the diet. The lack of differentially expressed detoxifying enzymes could also be due to overlaps of toxic chemistry on the three host plants.

With the exception of cytochrome P450s and GSTs not much is known about detoxifying enzymes applied by generalist insects. Hence such expressed enzymes could not be identified in databases. In the midgut we identified 4 unknown differentially expressed genes, which could very well be unknown genes involved in detoxification. We could, however, identify a differentially expressed gene homologous to a cytochrome P450 of *P. xylostella *in the restbody of *P. c-album *larvae. Cytochrome P450's are also involved in the metabolism of many endogenous compounds, hence this P450 expressed in the restbody is possibly not involved in the detoxification of plant defense compounds, but in metabolism.

While the detoxifying mechanisms appear not to be differentially expressed we see genes belonging to other classes differentially expressed. Short-chain dehydrogenases (SCDH) for example are upregulated in the midguts of on *Urtica *and *Ulmus *feeding larvae. SCDH form a large protein family with highly different enzymes, which only share 15-30% identity among each other [35]. They are present in all the life forms studied so far, have a very wide substrate spectrum and are generally involved in cellular differentiation and signaling [36].

Not surprisingly, also many digestive enzymes are differentially expressed in the midgut (Additional File 1; Table S1). For example an alpha-amylase was down-regulated in both tree diets in comparison to the stinging nettle. Amylases are enzymes participating in carbohydrate digestion. It is known that insects possess different amylases for starches [37]. It is possible that the differential expression we observe is due to different starch contents in the stinging nettle. We also found several serine proteases being differentially expressed upon feeding on the host plants, namely trypsins and chymotrypsins. Plants possess proteinase inhibitors (PIs), which are insect inducible peptidases that can suppress insect proteinases and by that reduce the digestibility [38,39]. It has been shown that lepidopteran larvae adapt their proteinases expression profile to the PI content of their food plant, upregulating proteinases that are insensitive to the plant PIs [37]. Our expression patterns suggest different proteinase requirements for the three host plants. We also excised four bands that were identified as homologous to Bmlipase-1; three of them were highly expressed in larvae feeding on *Urtica *and one on *Salix*. Insect midgut lipases have been studied in few insects so far and little to nothing is known on different expression of lipases in insects [37]. They form in insects a gene family that underwent many duplication events [40] with resulting diverse and overlapping function. Bmlipase-1 from *Bombyx mori *shows a high antiviral activity against *Bombyx mori *nucleopolyhedrovirus, although it is not inducible by viral infection. Its main function is probably as a digestive enzyme, as it is exclusively expressed in the gut tissue and has lipase activity [41].

We also found many ribosomal proteins to be differentially regulated upon feeding on different host plants, namely seven in the midgut and twelve in the restbody of *P. c-album*. Ribosomal genes are considered to be stably expressed and have been suggested and used as housekeeping genes in expression analysis. However, there are a number of ribosomal proteins that have been found to be differentially expressed upon different treatment and in different tissues [42], suggesting that there are major differences in expression patterns between different ribosomal proteins.

The complex pattern found in this study should perhaps not be surprising, considering that secondary metabolites, leaf texture and nutrient contents vary greatly between different plant species, and also depending on the plant age [43]. The three chosen *P. c-album *host plants have different leaf architecture, with the stinging nettle possessing thin hairy leaves, and both trees waxy leaves, that differ in shape. Besides the prominent stinging trichomes that only provide an effective defense against vertebrate herbivores [44], stinging nettle leaves also contain the phenolic compound caffeic acid, tannins, nicotine in measurable amounts and flavonoid glucosides [45,46]. *Salix *leaves has low levels of toxins but more condensed tannins [47], which is similar to the situation in elm [45,46].

Phenolic compounds, nicotine, tannins and flavonoids have been shown to affect insects feeding on them. Tannins for example are astringent, bitter plant polyphenols that either bind and precipitate or shrink proteins. Studies on the effect of tannins on the gut pH and redox potential of larvae found that many specialist and generalist insects have developed adaptations to cope with them [48,49]. Tannin metabolites are for example oxidized during the gut passage in the aquatic caterpillar *Acentria ephemerella*. Resistance against polyphenols appears to be correlated in general with better repair mechanisms in the gut tissue that enables to cope with free radical stress occurring during oxidation better [50]. Nicotine acts as agonist of the postsynaptic nicotinic acetylcholine receptors of the insect central nervous system. Metabolism of nicotine has been attributed to the action of cytochrome P450 [51,52]. Flavonoids in contrast can be sequestered from the diet and used for protection and pigmentation. In *B. mori *for example three flavonoid glycosides could be isolated from the cocoon shell [53]. The role of these secondary plant compounds in the interaction between *P. c-album *and its host plant and the adaptations *P. c-album *developed to them however, is not known.

Different plants also possess different microfloras [54], which affect immune response-related gene expression in the midgut of lepidopteran larvae [55]. We detected cobatoxin and gloverin homologous genes that are known to be inducible by bacterial challenge to be differentially expressed in the midgut tissue [56-58]. We see gloverin expression only in *Urtica *fed larvae and cobatoxin in *Salix *fed larvae, suggesting different microbial environments on those two species. In addition to differences in plant secondary metabolites *P c.-album *must therefore also face different bacterial quantities and qualities on its various host plants.

## Conclusion

Our data suggest a complex picture of gene expression in response to host plants, but also revealed some general patterns. While each plant evidently requires a unique gene regulation in the caterpillar, both phylogenetic relatedness and host plant growth form appear to influence the expression profile of the polyphagous comma butterfly, in agreement with phylogenetic studies of host plant utilization in butterflies.

## Methods

### Larval rearing and preparation

Stock used in the experiments was the offspring of four female comma butterflies collected in early May 2007 in the near-Stockholm area of Sweden. The females had already mated in the wild with unknown males and were put into cages for oviposition. Each female was presented with the host plants stinging nettle (*U. dioica*) and Great Sallow (*S. caprea*). Eggs were counted in the beginning of each day and were incubated in small jars on a sun-lit windowsill until hatching.

Larvae of each female were evenly spread across the three host plants. They were raised on stinging nettle, great sallow or elm (*U. glabra*) in individual jars. Plant material was cut from mature foliage of naturally growing plants and placed in water culture. The jars were placed in a climate room (temperature 20°C, LD 12:12) where larvae were raised to the 4th instar before dissection of the midgut. Plants were changed when needed due to withering or feeding. To maintain humidity, water was sprayed over the jars twice a day. Jars were changed randomly to avoid position effects. Between 10 and 43 individuals from each family were dissected, for a total of 109 individuals across the three different diets. Midguts and the rest of the larval body were preserved separately in RNA *later*^® ^(Ambion).

### RNA Isolation and Reverse Transcription

Larvae were dissected, and the midguts and restbodies were stored in RNA *later*^® ^(Ambion). Tissue samples were pooled (10-13 individuals) according to the larval diet (*Salix*, *Urtica*, *Ulmus*). The guts were homogenized by an Ultra-Turrax homogenizer (Beckman Coulter Scientific) in TRIzol (Invitrogen) reagent and restbodies were crushed in liquid nitrogen. For all samples TRIzol Reagent was used to isolate the RNA according to the manufacturer's protocol with the following modifications. After adding chloroform to separate the phases, the tubes were stored for 15 minutes at 4°C before centrifugation. To precipitate the RNA the solution was stored at -20°C overnight. After precipitation the RNA solution was centrifuged for 30 min at 4°C. The obtained dried pellet was dissolved in 90 μl RNA storage solution (Ambion), and any remaining genomic DNA contamination was removed by DNAse treatment (TURBO DNAse, Ambion). The DNAse enzyme was removed and the RNA was further purified by using the RNeasy MinElute Clean up Kit (Qiagen) following the manufacturer's protocol and eluted in 20 μl of RNA storage solution (Ambion).

### Differential gene expression

The GeneFishing (SeeGene) DEG Premix Kit essentially allows the amplification of the same set of genes from different samples due to a 10-mer core of arbitrary annealing control primers. By not exceeding the exponential phase of the PCR amplification, differentially expressed genes can be identified on an agarose gel.

To study differential gene expression between *P. c-album *larvae grown on different plants (*U*. glabra, *S*. *caprea *and *U*. *dioica*) the DEG GeneFishing Kit was used, following the manufacturer's protocol. In short 3 μg of DNA-free total RNA was converted into single-stranded cDNA using annealing control primer one (dTACP1) and a mixture of different reverse transcriptases (Array Script, Ambion; Power Script, Clontech; Bioscript, Bioline). Second-strand cDNA synthesis and subsequent PCRs were performed essentially as described in the DEG GeneFishing protocol. PCR products were separated and visualized on a 2% agarose gel. Differentially expressed bands were cut out from the agarose gels and PCR products extracted using Zymoclean Gel DNA Recovery Kit™ (Zymo Research) according to the manufacturer's instructions. DNA fragments were cloned into the pCR II TOPO vector (Invitrogen). Eight clones were picked for each extracted band and further processed.

### DNA Sequencing and Analysis

Plasmid minipreparations from bacterial colonies grown in 96 deep-well plates were performed using the 96 robot plasmid isolation kit (Eppendorf) on a Tecan Evo Freedom 150 robotic platform (Tecan). Single-pass sequencing of the 5' and 3' termini of individual clones was carried out on an ABI 3730 xl automatic DNA sequencer (PE Applied Biosystems).

Vector clipping, quality trimming and sequence assembly was done with the Lasergene software package (DNAStar Inc.). BLAST searches were conducted on a local server using the National Center for Biotechnology Information (NCBI) blastall program and best hits were recorded. Identified sequences of this study were submitted to Genbank and can be found under Accession numbers GT154770 - GT155229.

When two independently indentified differentially expressed sequences clustered in the assembly in the same contig, it was assumed to be a recent duplication.

### Quantitative real-time PCR

500 ng of DNA-free total RNA was converted into single-stranded DNA using a mix of random and oligo-dT20 primers according to the ABgene protocol (ABgene). Real-time PCR oligonucleotide primers were designed using the online Primer3 internet based interface . Primers were designed by the rules of highest maximum efficiency and sensitivity rules were followed to avoid formation of self and hetero-dimers, hairpins and self-complementarity. Gene-specific primers were designed on the basis of sequences obtained for selected *P. c-album *genes and two additional genes as potential housekeeping genes (ribosomal protein subunit 18S and elongation initiation factor 4) to serve as the endogenous control (normalizer) [see Additional file 2].

Both house-keeping primers were thoroughly tested. RPS 18 was the most consistent gene, and this was then used for the further analysis. QRT-PCR was done in optical 96-well plates on a MX3000P Real-Time PCR Detection System (Stratagene) using the Absolute QPCR SYBR green Mix (ABgene) to monitor double-stranded DNA synthesis in combination with ROX as a passive reference dye included in the PCR master mix.

### Scoring of expression similarities

Similarities in gene regulation were scored by counting upregulation (+/++) versus no expression (-). A semi-quantitative measure of upregulation was obtained by visually differentiating between strong (++) and weak (+) bands. The number of genes that were uniquely upregulated in any of the three plants, and the number of genes that were similarly upregulated in a pair of plants were then compared and analyzed with χ^2 ^goodness-of-fit tests (Figure 1 and 2).

## Authors' contributions

DF and HH-F carried out the molecular laboratory work, with the help of HV, and performed the statistical analysis of the data with the help of NJ and SN. LS carried out the qRT-PCR experiments with the help of DF and HH-F. LS, SN and NJ set up the study and reared larvae. All authors contributed in the conception and design of the study. HH-F, DH and NJ participated in drafting the manuscript. All authors participated in the writing and approval of the final manuscript.

## Supplementary Material

Additional file 1 **Supplemental Tables**. Supplemental Tables S1 - S3. Click here for file

Additional file 2 **qRT-PCR Primers**. List of primers used in the quantitative real-time PCR analysis. Click here for file
