# Supplementary material for: Phylogenetic relatedness and host plant growth form influence gene expression of the polyphagous comma butterfly (Polygonia c-album)
Source: BMC Genomics. 2009 Oct 31;10:506. doi: 10.1186/1471-2164-10-506 (PMC2775755; doi:10.1186/1471-2164-10-506)
Supplement: Additional file 1 — Supplemental Tables. Supplemental Tables S1 - S3. [file 1471-2164-10-506-S1.doc]

# Tables

## Table S1 - Differentially expressed genes in the larval midgut

| **Accession No.** | **Hit** | **e value** | **Salix** | **Urtica** | **Ulmus** |
| --- | --- | --- | --- | --- | --- |
|  | ***Digestion*** |  |  |  |  |
| GT154809 | chymotrypsin-like protease [*Helicoverpa armigera*], CAA72952.1 | 9.00E-84 | ++ | + | + |
| GT154892 | chymotrypsinogen-like protein 3 [*Manduca sexta*], CAM84318.1 | 3.00E-35 | - | ++ | - |
| GT154784 | trypsin Ia precursor [*Sesamia nonagrioides*], AAT95347.1 | 7.00E-20 | ++ | - | - |
| GT154811 | trypsin-like protease [*Helicoverpa armigera*], CAA72955.1 | 3.00E-37 | + | ++ | + |
| GT154832 | trypsin-like serine protease [*Ostrinia nubilalis*], AAX62033.1 | 8.00E-36 | + | + | ++ |
| GT154799 | RE38869p, alpha-amylase [*Drosophila melanogaster*], AAL48973.1 | 2.00E-97 | - | + | - |
| GT154851 | lipase-1 [*Bombyx mori*], NP_001036966.1 | 3.00E-57 | + | - | - |
| GT154781 | lipase-1 [*Bombyx mori*], NP_001036966.1 | 4.00E-62 | - | ++ | + |
| GT154804 | lipase [*Bombyx mandarina*], AAX39410.1 | 9.00E-49 | + | - | - |
| GT154783 | lipase-1 [*Bombyx mori*], NP_001036966.1 | 3.86E-48 | - | ++ | + |
| GT154783 | lipase-1 [*Bombyx mori*], NP_001036966.1 | 3.86E-48 | - | + | + |
| GT154924 | beta-glucosidase precursor [*Spodoptera frugiperda*], AAC06038 | 6.00E-04 | - | ++ | + |
| GT154808 | alpha-amylase 3 [*Diatraea saccharalis*], AAP97394.1 | 7.00E-26 | ++ | + | + |
| GT154837 | serine protease precursor [*Bombyx mori*], NP_001036826.1 | 3.00E-61 | ++ | + | + |
| GT154770 | serine protease [*Bombyx mandarina*], AAX39408.1 | 1.00E-18 | + | ++ | + |
| GT154837 | serine protease precursor [*Bombyx mori*], NP_001036826.1 | 3.00E-61 | + | ++ | + |
| GT154867 | serine protease [*Bombyx mori*], AAX39409.1 | 2.06E-05 | ++ | + | + |
| GT154904 | 35kDa protease [*Bombyx mori*], NP_001037037.1 | 3.00E-24 | - | ++ | - |
| GT154796 | zinc carboxypeptidase A 1 [*Culex pipiens quinquefasciatus*], XP_001851495.1 | 1.00E-65 | - | - | ++ |
| GT154796 | zinc carboxypeptidase A 1 [*Culex pipiens quinquefasciatus*], XP_001851495.1 | 1.00E-65 | + | + | ++ |
|  | ***Immunity*** |  |  |  |  |
| GT154789 | immune related protein [*Spodoptera frugiperda*], AAZ94260.1 | 3.60E-01 | - | + | + |
| GT154902 | cobatoxin long form B [*Spodoptera frugiperda*], AAQ18900.1 | 2.70E-01 | ++ | - | - |
| GT154780 | gloverin [*Trichoplusia ni*], ABV68856.1 | 6.00E-11 | - | ++ | - |
|  | ***Metabolism*** |  |  |  |  |
| GT154909 | proteasome 26S non-ATPase subunit 9 [*Bombyx mori*], NP_001093084.1 | 4.00E-44 | ++ | + | + |
| GT154909 | proteasome 26S non-ATPase subunit 9 [*Bombyx mori*], NP_001093084.1 | 4.00E-44 | ++ | + | ++ |
| GT154782 | PREDICTED: similar to CG3609-PA, oxidoreductase [*Apis mellifera*], XP_624408.1 | 3.00E-93 | - | ++ | - |
| GT154921 | NADH dehydrogenase subunit 1 [*Himantopterus dohertyi*], CAH59762 | 3.00E-10 | ++ | + | ++ |
| GT154863 | short-chain dehydrogenease/reductase 2 [*Bombyx mori*], NP_001040155.1 | 2.00E-03 | - | ++ | + |
| GT154782 | PREDICTED: similar to myo-inositol dehydrogenase [*Nasonia vitripennis*], XP_001603982.1 | 2.74E-91 | ++ | + | + |
| GT154873 | PREDICTED:similar to short-chain dehydrogenase [*Tribolium castaneum*], XP_001812912.1 | 8.00E-36 | ++ | ++ | + |
| GT154849 | PREDICTED: similar to tafazzin CG8766-PA, isoform A [*Apis mellifera*], XP_623296.1 | 4.00E-27 | ++ | + | + |
| GT154864 | peripheral-type benzodiazepine receptor [*Bombyx mori*], NP_001040343.1 | 9.00E-09 | + | ++ | ++ |
| GT154775 | selenoprotein M [*Litopenaeus vannamei*], ABI93178.1 | 2.63E-18 | + | ++ | + |
| GT154796 | zinc carboxypeptidase A 1 [*Culex pipiens quinquefasciatus*], XP_001851495.1 | 1.00E-65 | ++ | + | + |
| GT154805 | PREDICTED: similar to GM14009p, Long-chain acyl-CoA synthetases (AMP-forming) [*Nasonia vitripennis*], XP_001606071.1 | 1.42E-95 | - | ++ | - |
| GT154910 | PREDICTED: hypothetical protein, S-adenosyl-L-homocysteine hydrolase [*Nasonia vitripennis*], XP_001599389.1 | 2.00E-38 | + | ++ | ++ |
| GT154917 | conserved hypothetical protein, Glycosyl transferase family 2 [*Culex pipiens quinquefasciatus*], XP_001861763.1 | 3.00E-62 | ++ | + | + |
|  | ***Ribosomal proteins*** |  |  |  |  |
| GT154834 | ribosomal protein L39 [*Bombyx mori*], NP_001037251.1 | 8.17E-23 | ++ | - | ++ |
| GT154906 | ribosomal protein L27A [*Bombyx mori*]NP_001037522.1 | 6.72E-56 | + | ++ | + |
| GT154861 | ribosomal protein S16 [*Bombyx mori*], |NP_001037508.1 | 6.45E-34 | + | ++ | + |
| GT154819 | ribosomal protein S25 [*Bombyx mori*] NP_001037275.1 | 1.06E-38 | - | ++ | - |
| GT154813 | ribosomal protein L15 [*Bombyx mori*],NP_001037162.1 | 1.06E-38 | + | - | + |
| GT154812 | ribosomal protein S2 [*Bombyx mori*], NP_001037564.1 | 1.00E-109 | + | ++ | + |
|  | ***Translation regulation/DNA Structure*** |  |  |  |  |
| GT154900 | hypothetical protein AaeL_AAEL004467, similar to Chromobox protein [*Aedes aegypti*], XP_001649228.1 | 2.00E-04 | ++ | - | - |
| GT154826 | eukaryotic initiation factor 5A [*Papilio xuthus*], BAG30779.1 | 2.00E-41 | + | ++ | + |
| GT154888 | argonaute 2 [*Bombyx mori*], NP_001036995.1 | 7.05E-72 | ++ | + | ++ |
| GT154862 | Sui1 protein [*Bombyx mori*], NP_001037082.1 | 3.90E-16 | ++ | - | - |
| GT154802 | PREDICTED: similar to GA18560-PA , Predicted cysteine protease (OTU family)[Posttranslational modification, protein turnover, chaperones][*Nasonia vitripennis*], XP_001602110.1 | 1.71E-95 | + | + | ++ |
| GT154824 | PREDICTED: similar to chromosome 1 open reading frame 151 protein [*Tribolium castaneum*], XP_001808987.1 | 3.00E-03 | ++ | + | + |
|  | ***Transport*** |  |  |  |  |
| GT154920 | transmembrane emp24 protein transport domain containing 9 [*Bombyx mori*], NP_001040538.1 | 4.37E-104 | + | ++ | ++ |
|  | ***Unknown*** |  |  |  |  |
| GT154815 | AGAP003713-PA [*Anopheles gambiae* str. PEST], XP_001230950.2 | 1.10E-02 | - | - | + |
| GT154903 | AGAP003713-PA [*Anopheles gambiae* str. PEST], XP_001230950.2 | 5.00E-03 | - | - | ++ |
| GT154830 | unknown [*Helicoverpa armigera*], ABU98617.1 | 3.00E-07 | + | ++ | + |
| GT154871 | PREDICTED: similar to CG11964 CG11964-PA [*Tribolium castaneum*], XP_967379.1 | 5.00E-32 | + | ++ | + |
| GT154911 | PREDICTED: similar to DC2 protein [*Tribolium castaneum*], XP_001811714.1 | 4.00E-42 | + | - | + |

Identified differentially expressed genes identified from larvae fed on three different host plants (*Salix*, *Ulmus*, *Urtica*) from the GeneFishing experiments of the midguts of *P. c-album*. Shown are the accession numbers and the best BLAST hits with e-values for each gene. Differential gene expression visualized as band intensity on agarose gels is depicted (++ strong, + moderate, - absent).

## Table S2 - Differentially expressed genes in the larval restbody

| **Accession No** | **Hit** | **e value** | **Salix** | **Urtica** | **Ulmus** |
| --- | --- | --- | --- | --- | --- |
|
|  | ***Cell Signaling*** |  |  |  |  |
| GT155194 | Der1-like domain family member 1, [*Bombyx mori*], NP_001040297.1| | 5.7408E-100 | + | + | - |
|  | ***Detoxification*** |  |  |  |  |
| GT155053 | cytochrome P450, [*Plutella xylostella*], BAF95609.1| | 0.00000001 | ++ | + | ++ |
|  | ***Hormon synthesis*** |  |  |  |  |
| GT154997 | similar to CG10638-PA, [*Papilio xuthus*], BAG30781.1| | 1E-28 | ++ | ++ | + |
| GT155179 | juvenile hormone epoxide hydrolase, [*Bombyx mori*], BAF81491.1| | 1E-113 | ++ | + | + |
|  | ***Immunity*** |  |  |  |  |
| GT154966 | conserved hypothetical protein, [*Culex pipiens quinquefasciatus*], |XP_001869584.1 | 3E-36 | + | ++ | - |
| GT155132 | mitochondrial aldehyde dehydrogenase, [*Bombyx mori*], NP_001040198.1| | 7E-39 | + | + | - |
|  | ***Metabolism*** |  |  |  |  |
| GT155186 | mitochondrial aldehyde dehydrogenase, [*Bombyx mori*], NP_001040198.1| | 8E-50 | - | + | - |
| GT154933 | methionine-rich storage protein, [*Spodoptera exigua*], EU259816 | 4E-81 | + | ++ | ++ |
| GT154933 | methionine-rich storage protein, [*Spodoptera exigua*], ABX55887 | 4E-81 | ++ | + | + |
| GT154933 | methionine-rich storage protein, [*Spodoptera exigua*],ABX55887 | 4E-81 | + | - | ++ |
| GT155014 | aspartate aminotransferase, [*Bombyx mori*], NP_001040337.1| | 2E-35 | ++ | ++ | + |
| GT155085 | alpha-amylase, [*Helicoverpa armigera*], ABU98613.1| | 2E-77 | + | ++ | - |
| GT155081 | putative reverse transcriptase, [*Zingiber officinale*], ABK60177 | 1E-20 | + | ++ | + |
| GT154987 | vacuolar proton atpases [*Aedes aegypti*] XP_001657344.1 | 4.26E-24 | ++ | ++ | + |
| GT154976 | PREDICTED: similar to BcDNA.GH02921, [*Nasonia vitripennis*,] |XP_001600178.1| | 6E-53 | + | ++ | - |
| GT155009 | PREDICTED: similar to CG2656-PA, [*Apis mellifera*], XP_625026.1 :Conserved hypothetical ATP binding protein. | 6E-23 | ++ | ++ | + |
| GT154989 | cyclic beta 1-2 glucan synthetase [*Xanthomonas campestris pv. campestris* str. ATCC 33913] NP_637420.1 | 3.34E+00 | - | - | ++ |
| GT154986 | PREDICTED: similar to alpha isoform of regulatory subunit A, protein phosphatase 2, [*Apis mellifera*], XP_001120202.1| | 5.60577E-47 | + | ++ | + |
| GT154942 | hypothetical protein PFE0755c, [*Plasmodium falciparum* 3D7], XP_001351708.1|, NADH dehydrogenase subunit | 0.53 | ++ | ++ | + |
| GT154955 | trypsin, [*Choristoneura fumiferana*], AAA84423.1| | 1E-23 | ++ | ++ | + |
| GT155076; GT155057 | ubiquinol-cytochrome c reductase, [*Bombyx mori*], NP_001106738.1 | 0.00000007 | ++ | ++ | + |
| GT155053 | 26S proteasome regulatory ATPase subunit 10B, [*Bombyx mori*], NP_001040484 | 0.00004 | + | - | + |
| GT155208 | serine protease precursor, [*Bombyx mori*],NP_001036826.1| | 3E-50 | ++ | ++ | + |
| GT154946 | serine protease precursor [*Bombyx mori*], NP_001036826.1 |  | - | + | - |
| GT155225 | phosphate transport protein [*Bombyx mori* ] | 6.00E-104 | - | + | - |
|  | ***Ribosomal protein*** |  |  |  |  |
| GT154953 | ribosomal protein S7 [*Bombyx mori*] NP_001037261.1 | 4.12E-67 | + | + | - |
| GT155067 | ribosomal protein L35A, [*Bombyx mori*], NP_001037243.1 | 2E-37 | ++ | ++ | + |
| GT154950 | ribosomal protein S11-1 [*Bombyx mori*] AAV34867.1 | 7.47E-37 | ++ | ++ | + |
| GT154977 | ribosomal protein S25 [*Bombyx mori*] NP_001037275.1 | 1.27E-31 | - | + | - |
| GT154940 | ribosomal protein S10 [*Bombyx mori*] NP_001037524.1 | 2.42E-51 | ++ | + | + |
| GT154940 | ribosomal protein S10 [*Bombyx mori*] NP_001037524.1 | 2.42E-51 | + | ++ | + |
| GT154958 | ribosomal protein L39, [*Bombyx mori*], NP_001037251.1 | 9E-23 | ++ | + | + |
| GT155224 | ribosomal protein L5, [*Bombyx mori*], AAV34814 | 5E-76 | + | + | - |
| GT155195 | ribosomal protein L27 [*Bombyx mori*] NP_001037235.1 | 2.91E-68 | + | ++ | + |
| GT155222 | ribosomal protein L6, [*Bombyx mori*], NP_001037132.1 | 2E-26 | ++ | ++ | + |
| GT155214 | ribosomal protein L27A [*Bombyx mori*] NP_001037522.1 | 1.37E-55 | ++ | + | ++ |
| GT155192 | ribosomal protein S18 [*Bombyx mori*] NP_001037269.1 | 1.57E-42 | + | ++ | + |
|  | ***Silk production*** |  |  |  |  |
| GT154984 | BAB39503.1| fibroin L-chain [*Papilio xuthus*] | 1E-46 | + | ++ | - |
| GT154939 | fibroin L-chain, [*Papilio xuthus*], BAB39503.1| | 7E-47 | + | ++ | - |
|  | ***Stress*** |  |  |  |  |
| GT155980; GT155074 | heat shock cognate 70 protein, [*Sesamia nonagrioides*], AAY26452.2| | 2E-37 | + | + | ++ |
|  | ***Structure*** |  |  |  |  |
| GT155058 | Kettin1 protein, [*Helicoverpa armigera*],ABU96746.1| | 4E-91 | ++ | + | ++ |
| GT154949 | obstractor B, [*Tribolium castaneum*], NP_001073566 ,Chitin binding Peritrophin-A domain | 1E-104 | + | + | - |
| GT154954 | CU15_MANSE Cuticle protein CP14.6 precursor (MSCP14.6), Q94984| | 0.001 | ++ | ++ | + |
| GT154962 | cuticular protein CPR41A [*Papilio xuthus*], BAG30737.1 | 6E-36 | ++ | ++ | - |
| GT155226; GT154964; GT154940; GT155178 | cuticular protein 78, RR-1 family (AGAP009876-PA), [*Anopheles gambiae* str. PEST], XP_318996 | 1E-10 | ++ | ++ | + |
| GT155227 | pupal cuticle protein [*Bombyx mori*], NP_001119729 | 1.7 | + | ++ | - |
| GT155213 | mCG13192, isoform CRA_a, [*Mus musculus*], EDL05910.1| | 5.87055E-05 | + | + | - |
| GT155193 | basement membrane collagen,[*Brugia malayi*], AAC46611.1| | 2E-39 | + | + | - |
|  | ***Translation regulation/ Cell structrure*** |  |  |  |  |
| GT154948 | eukaryotic initiation factor 5A [*Papilio xuthus*], AB264704 | 2E-41 | ++ | ++ | + |
| GT154944 | histone H3.3 type 2, [*Culex pipiens quinquefasciatus*],XP_001865500 | 5E-43 | + | - | ++ |
| GT154943 | small nuclear ribonucleoprotein E, [*Bombyx mori*], NP_001040370.1| | 1E-20 | ++ | ++ | - |
| GT154961 | elongation factor 1 alpha, [*Papilio xuthus*],BAG30769 | 2E-52 | + | + | - |
| GT155045 | PREDICTED: similar to exosome complex exonuclease RRP41, putative, [*Tribolium castaneum*], XP_975230.2 | 8E-20 | ++ | ++ | - |
| GT155023 | ribophorin, [*Aedes aegypti*], XP_001663283.1| | 8E-69 | + | + | - |
| GT155229 | PREDICTED: similar to shroom family member 4, [*Danio rerio*],XP_687426 | 1.3 | - | ++ | - |
|  | ***Transport*** |  |  |  |  |
| GT154956 | binding-protein-dependent transport systems inner membrane component [*Roseiflexus sp*. RS-1], ABQ88845.1 | 0.231895 | + | + | ++ |
| GT154985 | binding-protein-dependent transport systems inner membrane component [*Roseiflexus sp*. RS-1], ABQ88845.1 | 2.28E-01 | + | - | + |
| GT154937 | sodium-dependent phosphate transporter, [*Aedes aegypti*], XP_001658313.1| | 3E-76 | + | - | + |
| GT155191 | sodium-dependent phosphate transporter, [*Aedes aegypti*],XP_001658313.1| | 6E-76 | ++ | ++ | + |
| GT154938 | signal sequence receptor beta subunit, [*Bombyx mori*], NP_001040332.1| | 2E-21 | ++ | ++ | + |
| GT155112 | transport protein Sec61 alpha subunit, [*Bombyx mori*], NP_001037628.1 | 0.79 | ++ | - | - |
|  | ***Unknown*** |  |  |  |  |
| GT154973 | hypothetical protein, [*Paramecium tetraurelia*], XP_001428456.1| | 3.9 | ++ | ++ | + |
| GT154936 | hypothetical protein UM00309.1, [*Ustilago maydis* 521], XP_756456 | 0.46 | ++ | ++ | - |
| GT154935 | PREDICTED: hypothetical protein [*Homo sapiens*], XP_001714781 | 2.6 | + | + | ++ |
| GT154971 | unknown [*Drosophila pseudoobscura pseudoobscura*] XM_002133986 | 0.041 | + | - | ++ |

Identified differentially expressed genes for larvae fed on three different plants (Salix, Ulmus, Urtica) from the GeneFishing experiments of the restbodies of *P. c-album*. Shown are the accession numbers and the best BLAST hits with e-values for each gene. Differential gene expression visualized as band intensity on agarose gels is depicted (++ strong, + moderate, - absent).

## Table S3 - qRT-PCR results for *P. c-album* midguts and restbodies

| **Accession No** | **relative fold gene expression** | | **match with Gene Fishing data** | | | **best BLAST hit** |
| --- | --- | --- | --- | --- | --- | --- |
|  | **Salix** | **Ulmus** | **Urtica-Ulmus** | **Salix-Ulmus** | **Salix-Urtica** |  |
| ***Midguts*** | | |  |  |  |  |
| GT154892 | **-7.79 ± 0.62** | **-2.90 ± 0.28** |  |  |  | gi|146327862|emb|CAM84318.1| chymotrypsinogen-like protein 3 [*Manduca sexta*] |
| GT154861 | -1.18 ± 0.15 | **-2.58 ± 0.24** |  |  |  | gi|112984394|ref|NP_001037508.1| ribosomal protein S16 [*Bombyx mori*] |
| GT154786 | -1.29 ± 0.11 | 1.36 ± 0.08 |  |  |  | gi|112983142|ref|NP_001037037.1| 35kDa protease [*Bombyx mori*] |
| GT154805 | **-2.03 ± 0.01** | **-2.32 ± 0.15** |  |  |  | gi|156550737|ref|XP_001606071.1| PREDICTED: similar to GM14009p [*Nasonia vitripennis*] |
| GT154813 | 1.27 ± 0.16 | 1.09 ± 0.04 |  |  |  | gi|54609223|gb|AAV34827.1| ribosomal protein L15 [*Bombyx mori*] |
| GT154850 | 1.02 ± 0.02 | **-2.18 ± 0.03** |  |  |  | gi|112983352|ref|NP_001036966.1| lipase-1 [*Bombyx mori*] |
| GT154928 | **-6.78 ± 0.06** | **-6.14 ± 0.04** |  |  |  | gi:61191881 serine protease [*Bombyx mandarina*] |
| GT154809 | **2.22 ± 0.04** | -1.34 ± 0.04 |  |  |  | gi|2463064|emb|CAA72952.1| chymotrypsin-like protease [*Helicoverpa armigera*] |
| GT154825 | **-4.76 ± 0.03** | -1.87 ± 0.002 |  |  |  | gi|2463070|emb|CAA72955.1| trypsin-like protease [*Helicoverpa armigera*] |
| GT154834 | -1.09 ± 0.05 | -1.16 ± 0.15 |  |  |  | gi|112984118|ref|NP_001037251.1| ribosomal protein L39 [*Bombyx mori*] |
| GT154902 | -1.47 ± 0.06 | **-6.76 ± 0.03** |  |  |  | gi:33439724] cobatoxin long form B [*Spodoptera frugiperda*] |
| GT154920 | -1.55 ± 0.04 | -1.37 ± 0.03 |  |  |  | gi|114052711|ref|NP_001040538.1| transmembrane emp24 protein transport domain containing 9 [*Bombyx mori*] |
| GT154797 | **-4.14 ± 0.06** | -1.15 ± 0.02 |  |  |  | gi|108881060|gb|EAT45285.1| zinc carboxypeptidase [*Aedes aegypti*] |
| GT154862 | -1.18 ± 0.04 | -1.48 ± 0.04 |  |  |  | gi|112983000|ref|NP_001037082.1| Sui1 protein [*Bombyx mori*] |
| GT154782 | 1.69 ± 0.06 | -0.54 ± 0.04 |  |  |  | gi|66514540|ref|XP_624408.1| PREDICTED: similar to CG3609-PA [*Apis mellifera*] |
| GT154799 | **-2.12 ± 0.02** | -1.79 ± 0.03 |  |  |  | gi|157126491|ref|XP_001660906.1| alpha-amylase [*Aedes aegypti*] |
| GT154861 | -1.50 ± 0.06 | 1.10 ± 0.02 |  |  |  | gi|114050773|ref|NP_001040155.1| short-chain dehydrogenease/reductase 2 [*Bombyx mori*] |
| GT154866 | **10.02 ± 0.05** | 1.56 ± 0.13 |  |  |  | gi|157113343|ref|XP_001657786.1| trypsin [*Aedes aegypti*] |
| ***Restbodies*** | | |  |  |  |  |
| GT154962 | -1.65 ± 0.04 | **-3.02 ± 0.00** |  |  |  | cuticular protein CPR41A [*Papilio xuthus*], gi:183979370 |
| GT154949 | -1.54 ± 0.20 | -1.01 ± 0.04 |  |  |  | obstractor B, [*Tribolium castaneum*], gi:121582324,Chitin binding Peritrophin-A domain |
| GT154942 | -1.22 ± 0.15 | 1.14 ± 0.04 |  |  |  | hypothetical protein PFE0755c, [*Plasmodium falciparum* 3D7], gi:124506221, NADH dehydrogenase subunit |
| GT155085 | **-8.31 ± 0.05** | **-16.68 ± 0.25** |  |  |  | alpha-amylase, [*Helicoverpa armigera*], gb|ABU98613.1| |
| GT154966 | **-2.11±** | -1.04 ± |  |  |  | ref|XP_001869584.1| conserved hypothetical protein, Destabilase [*Culex pipiens quinquefasciatus*] 3E-36 |
| GT155053 | 1.27 ± 0.01 | **2.36 ± 0.14** |  |  |  | cytochrome P450, [*Plutella xylostella*], dbj|BAF95609.1| |
| GT154946 | **-33.24 ± 0.44** | **-9.45 ± 0.04** |  |  |  | serine protease precursor [*Bombyx mori*], NP_001036826.1 |
| GT154980 | -1.38 ± 0.04 | **2.04 ± 0.11** |  |  |  | gi|157064217|gb|AAY26452.2| heat shock cognate 70 protein [*Sesamia nonagrioides*] |
| GT154984 | -1.90 ± 0.44 | 1.04 ± 0.25 |  |  |  | gi|13383201|dbj|BAB39503.1| fibroin L-chain [*Papilio xuthus*] |

qRT-PCR results for *P. c-album* midguts and restbodies fed on three different plants (Salix, Urtica, Ulmus). Relative expression of genes of interest were normalized using RPS18 as an expression control. The gene expression of larvae fed on Urtica was used as a reference to which relative expression in larvae fed on Ulmus and Salix was compared to (values are mean ± SD). Consistency with GeneFishing results is depicted in color-code. (black bars – agreement, white – disagreement). Compared are always two diets and their relative expression to each other.
