# Supplementary material for: Phylogenetic relatedness and host plant growth form influence gene expression of the polyphagous comma butterfly (Polygonia c-album)
Source: BMC Genomics. 2009 Oct 31;10:506. doi: 10.1186/1471-2164-10-506 (PMC2775755; doi:10.1186/1471-2164-10-506)
Supplement: Additional file 2 — qRT-PCR Primers. List of primers used in the quantitative real-time PCR analysis. [file 1471-2164-10-506-S2.pdf]

Gene	Forward primer	Reverse primer
Midguts		
MC 123	ATCAGAGCTCCAGCTGTTCAA	CCACGACACTACTCCGATTTG
MC 92	GATTTCTGGTATCTGGCACGA	ACGCCATCAGACAAGCTATTT
MC 17	ACAACCATACCGACCAATCTG	CAGTAGCTGGTGCATCCTCTC
MC 36	TCTTTGGTACGGACAATCTCG	ATACGCTGTGCTTATCGGAGA
MC 44	CATCGCTTCTCTCAAACCAAG	CCACTGGCTTTCCATCTATGA
MC 81	CGATCCAATCTCTAACGCTGA	TACGAAGCTGGTTACCATTGC
MC 159	CTCTGATCCAGGCGTTGAAT	GGCACTCTGATCGGTATCGTA
MC 40	CATTGGTTATCACGCTGAGGT	CGCATTCCTGCAGTAACCTAC
MC 42	TTGGACCGTGAACCAGAATAC	GTTGCTGCTAAACGAGACGAC
MC 65	CGGCCCACAAGACATTTATTA	AGCTTGAGCTTGGTTCTCCTC
MC 133	CAGCGACCGAATCTGAAGTTA	AGTTAGCCCGACCAGAGCTT
MC 151	TATGAGGACACCGGTTTGAAG	TGACAGAACTGCAATTGAGGA
MC 28	CCATTTATACCGCAACAGGAG	TGGAAGTAGGAAACCGAACTG
MC 93	AACGCGAGAACATTTGTCAGT	ACATGTTACGTGTCGGTGATG
MC 13	CAAACAGGCGTCAAACTCAAT	ACTGGTCACGTCTTGTGGAAC
MC 30	GTTTACCCAGACGAGCCTACC	CGCATAGACTTCGGTCATCAT
MC 94	TCATATCGGCGATGATAGACA	GGAACCGGAAGAAACCAAGAT
MC 97	GTAATCGTCGGTGTTGCAGTT	TACCCTTAGCCGACTGTCAGA
Restbodies		
RC 1	TTAATTCCAAGCCCACGTATG	GCTGGTATTGTCGATTTGCTC
RC 13	GCTTCTTTGCTGATGCTGAAC	TGTTGGTATCAGCAACAACCA
RC 2	ACGAAGGAAAGCGACGACTAT	GTCCTAGCAGCCACGTGTAAC
RC 4	TAAACTCCGACGGTTCTTGTG	TTGCCATTATCCCACCAGTTA
RC 43	GGTGTCGTGAACTGCTACGAT	GACAGTTGCTACGCACTGGTT
RC 44	CCTGGAAAGTATTGCGACAGA	TCAAGGTACGTGTCCAATTCC
RC 47	TGTTGGTATCAGCAACAACCA	GGAGGCAACAATGATACTGGA
RC 78	TTGGTCGTAAATTCGAGGATG	TCCTCAGGGAAGAAGGTCTTG
RC 9	AAAGCCAAGTCATGTTGCTACT	TCACTCGTTACCTTCACAACG
